# Cell Chirality Drives Left-Right Asymmetric Morphogenesis

**DOI:** 10.3389/fcell.2018.00034

**Published:** 2018-04-03

**Authors:** Mikiko Inaki, Takeshi Sasamura, Kenji Matsuno

**Affiliations:** Department of Biological Sciences, Graduate School of Science, Osaka University, Osaka, Japan

**Keywords:** cell chirality, left-right asymmetry, F-actin, Myosin I, *Drosophila*

## Abstract

Most macromolecules found in cells are chiral, meaning that they cannot be superimposed onto their mirror image. However, cells themselves can also be chiral, a subject that has received little attention until very recently. In our studies on the mechanisms of left-right (LR) asymmetric development in *Drosophila*, we discovered that cells can have an intrinsic chirality to their structure, and that this “cell chirality” is generally responsible for the LR asymmetric development of certain organs in this species. The actin cytoskeleton plays important roles in the formation of cell chirality. In addition, *Myosin31DF* (*Myo31DF*), which encodes *Drosophila* Myosin ID, was identified as a molecular switch for cell chirality. In other invertebrate species, including snails and *Caenorhabditis elegans*, chirality of the blastomeres, another type of cell chirality, determines the LR asymmetry of structures in the body. Thus, chirality at the cellular level may broadly contribute to LR asymmetric development in various invertebrate species. Recently, cell chirality was also reported for various vertebrate cultured cells, and studies suggested that cell chirality is evolutionarily conserved, including the essential role of the actin cytoskeleton. Although the biological roles of cell chirality in vertebrates remain unknown, it may control LR asymmetric development or other morphogenetic events. The investigation of cell chirality has just begun, and this new field should provide valuable new insights in biology and medicine.

## Introduction

The directional left-right (LR) asymmetry of body structures and functions is found in animals across phyla. In this review, directional LR asymmetry is called “LR asymmetry.” LR asymmetry is a fundamental property of animal development, and the mechanisms of LR asymmetric development are a topic of strong interest in various biological and medical fields. To explain the molecular basis of LR asymmetric development, Walport proposed the “F molecule” hypothesis (Brown and Wolpert, 1990). In this hypothesis, the F molecule is chiral and can be arranged along the anterior-posterior and dorsal-ventral axes. An object is chiral if it cannot be superposed onto its mirror image. By virtue of these properties, the F molecule can direct the LR axis based on its chirality. This idea is supported by findings on the molecular mechanisms of LR asymmetric development in mouse.

In mouse, motile cilia in the node, which is a small pit located in the ventral side of the early embryo, rotate clockwise and induce a leftward flow of extra-embryonic fluid, named the “nodal flow” (Nonaka et al., 1998). The nodal flow is the first cue that breaks the LR symmetry of the mouse embryo. The nodal flow induces left-side-specific gene expression, which leads to subsequent LR asymmetric development (Nonaka et al., 1998, 2002; Okada and Hirokawa, 1999). In addition to their ventral location, the nodal cilia slant posteriorly, which helps them to generate the leftward fluid flow (Nonaka et al., 2005). Thus, the nodal cilia are arranged in a polar manner along both the anterior-posterior and dorsal-ventral axes. The nodal cilia also have intrinsic chirality, because they rotate clockwise (Nonaka et al., 1998). Therefore, the nodal cilia satisfy the requirements of the F molecule (Brown and Wolpert, 1990). In this case, the molecular origin of LR asymmetric development is traced back to the chiral structure and motion of cilia, which is still a subject of active investigation (Okada and Hirokawa, 2009; Nonaka, 2013).

## Cell chirality drives LR asymmetric morphogenesis in *Drosophila*

**Figure 1:**
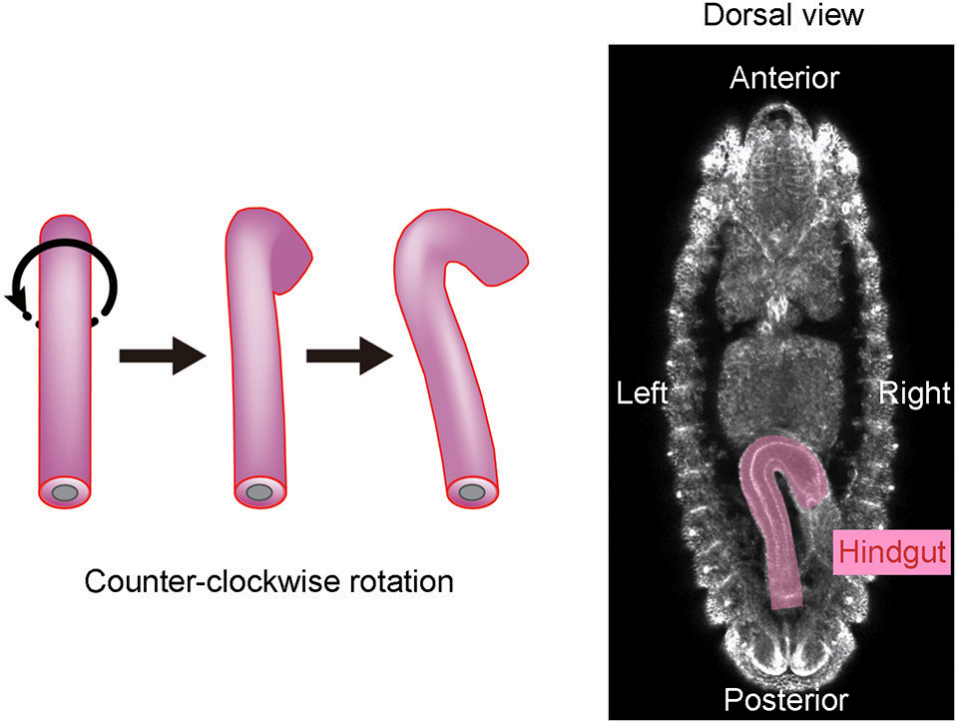
The *Drosophila* embryonic hindgut rotates 90° counterclockwise. **(Left)** The embryonic hindgut first forms as a bilaterally symmetric structure that curves ventrally **(Left)**. It rotates 90° counterclockwise from the posterior view **(Middle)**, and consequently curves to the right **(Right)**. **(Right)** The embryonic gut curves rightward at stage 12 in wild-type *Drosophila*. This figure is partly adapted from Inaki et al. (2016) with permission.

*Drosophila melanogaster* is another organism in which the mechanisms of LR asymmetric development have been extensively studied (Hozumi et al., 2006; Spéder et al., 2006; González-Morales et al., 2015; Inaki et al., 2016). In this organism, chirality at the level of cells, rather than molecules, was found to contribute to LR asymmetric development for the first time (Taniguchi et al., 2011; Inaki et al., 2016). The embryonic hindgut first forms LR symmetrically along the midline, then rotates counterclockwise 90° as viewed from the posterior, and eventually exhibits a LR asymmetric morphology (Figure 1). Before the rotation, the hindgut has a hook-like shape that points ventrally, and its most posterior part is stably connected to the anus (Figure 1). The hindgut then twists, causing the hook-like shape to point rightward (Figure 1).

**Figure 2:**
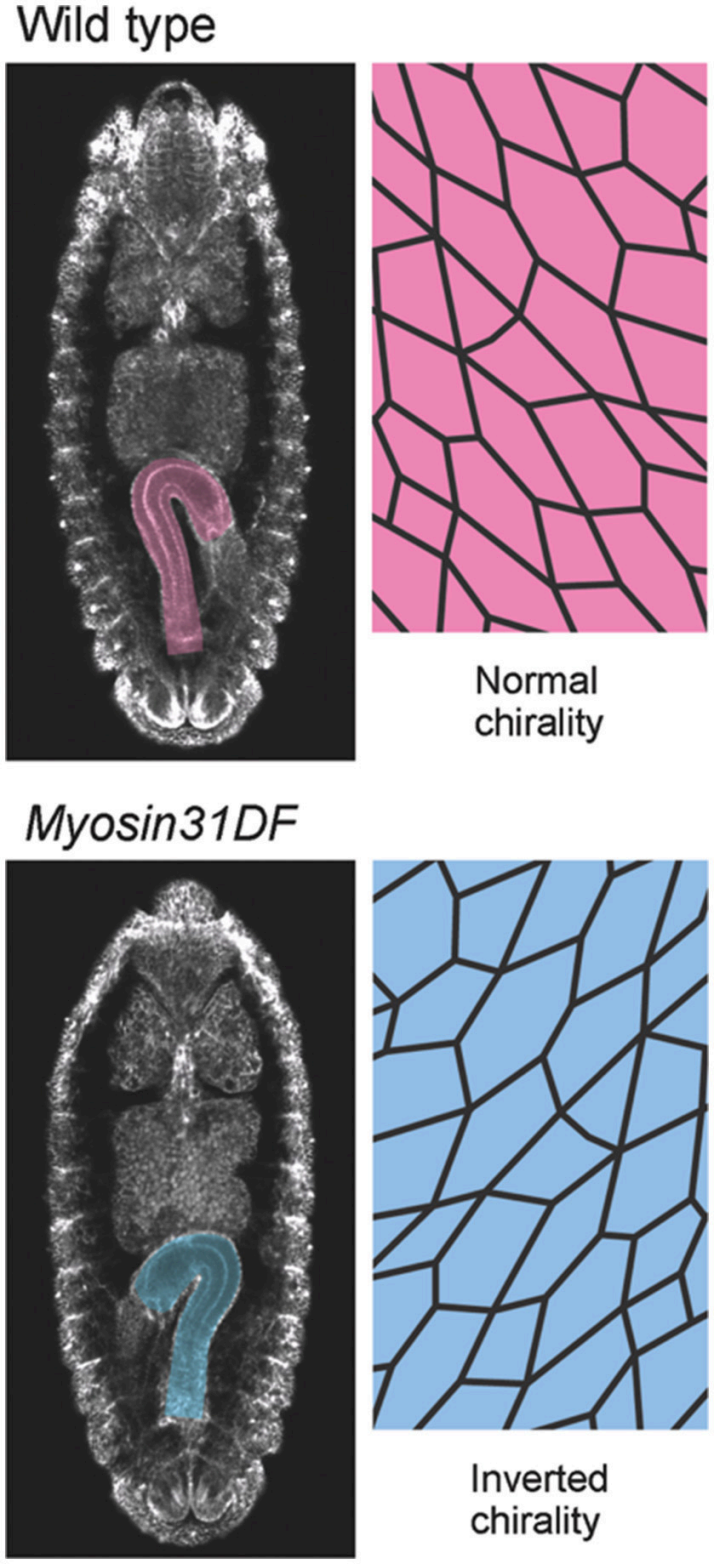
*Myosin31DF* mutation reverses the cell chirality and hindgut rotation. **(Top)** A wild-type *Drosophila* embryo shows normal cell chirality and a rightward-pointing hindgut. **(Bottom)** In the *Myosin31DF* mutant, the cell chirality and hindgut laterality are the mirror images of those in its wild-type counterpart. This figure is partly adapted from Inaki et al. (2016) with permission.

**Figure 3:**
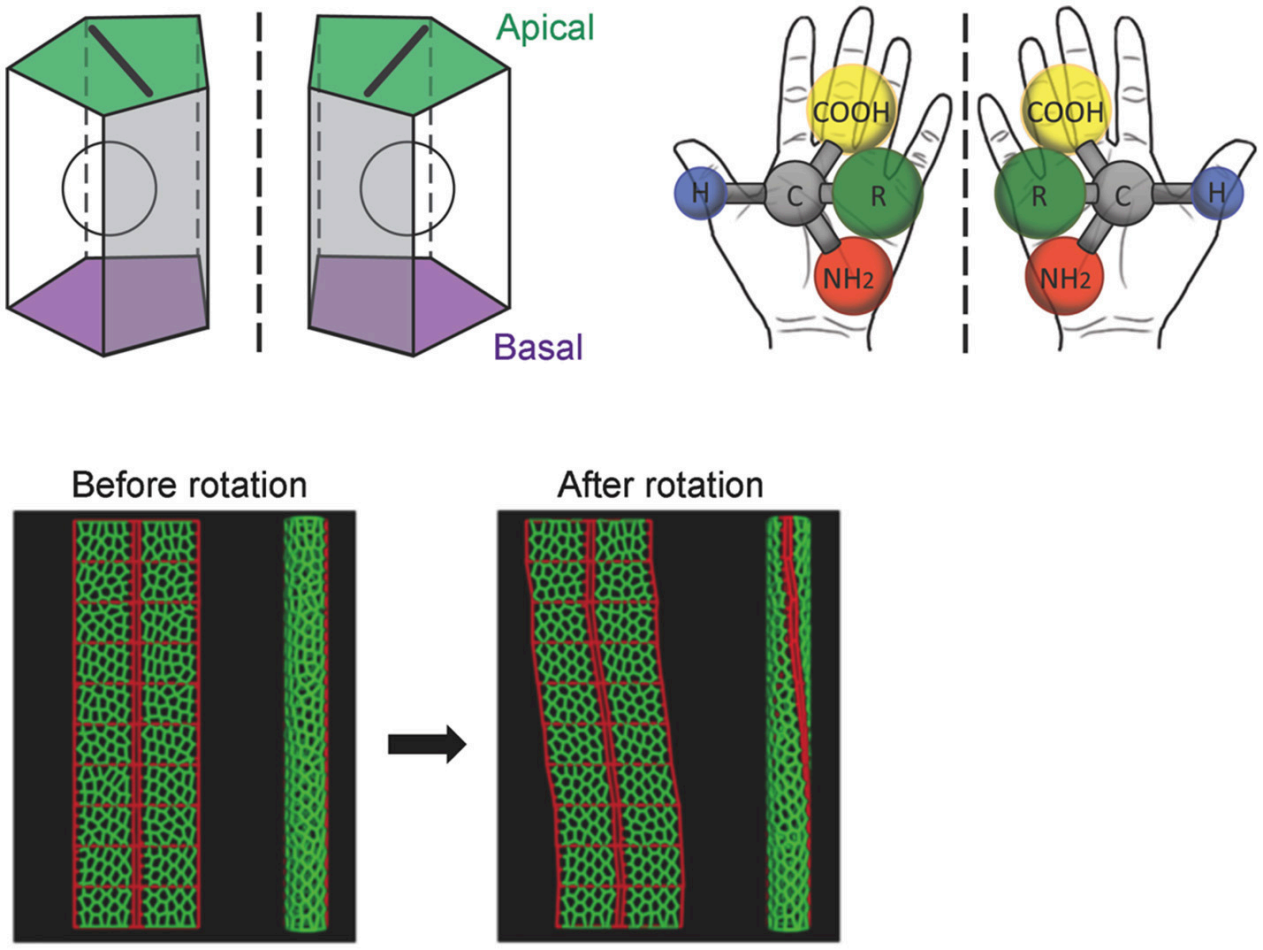
A computer simulation recapitulates the cell-chirality-driven counterclockwise rotation of the hindgut. **(Top)** The shape of the apical surface of hindgut epithelial cells is LR-asymmetric **(Left)**. Considering that these cells also have apical-basal polarity, they show chirality. This property is illustrated by left-handed and right-handed chiral amino acids **(Right)**. **(Bottom)** To test whether cell chirality alone could induce the axial rotation of the hindgut epithelial tube, a computer simulation based on a vertex model was performed. The introduction of LR bias to the contraction of the cell boundary was sufficient to recapitulate the cell chirality found *in vivo*. The introduction and subsequent release of cell chirality were sufficient to induce the LR-asymmetric rotation of the model epithelial tube (Taniguchi et al., 2011). These figures are partly adapted from Inaki et al. (2016) and Taniguchi et al. (2011) with permissions.

Before the rotation, the anterior-posterior axis direction of the hindgut epithelial tube can be readily defined and used as a reference in analyzing the LR asymmetry of each cell. Taniguchi et al. discovered that before rotation onset, the hindgut epithelial cells show LR asymmetry in their apical surface, which faces the lumen of the hindgut tube (Figure 2; Taniguchi et al., 2011). In these cells, leftward-tilting cell boundaries are found more frequently than rightward-tilting ones (Figure 2). In addition, the hindgut epithelial cells have apical-basal polarity, like other epithelial cells. Therefore, the three-dimensional hindgut epithelial cells are chiral, because their shape cannot be superimposed onto their mirror image (Figure 3). This property of cells was called “cell chirality” (Taniguchi et al., 2011; Inaki et al., 2016). The chirality of these cells eventually disappears as the rotation progresses, and the cell shape becomes achiral (LR symmetric) when the rotation is completed (Taniguchi et al., 2011).

A computer simulation demonstrated that the introduction and subsequent dissolution of cell chirality is sufficient to induce the counterclockwise rotation of a model epithelial tube (Taniguchi et al., 2011). The results of this model study were also consistent with previous observations that cell proliferation and cell death do not occur during hindgut rotation, suggesting that cell rearrangement and/or deformation alone is responsible for the hindgut rotation (Lengyel and Iwaki, 2002; Wells et al., 2013).

## *Myosin31DF* is a switch for cell chirality and LR asymmetry

Although the mechanisms of cell-chirality formation remain unclear, important clues emerged from the genetic identification of *Drosophila Myosin31DF* (*Myo31DF*) mutants, in which the LR asymmetry of various organs is reversed (Hozumi et al., 2006; Spéder et al., 2006). *Myo31DF* is the *Drosophila* ortholog of *Myosin1D*, which encodes an evolutionarily conserved class I myosin (Hozumi et al., 2006; Spéder et al., 2006). In loss-of-function *Myo31DF* mutants, the hindgut rotates 90° in the opposite direction to that of wild type in more than 80% of the flies, resulting in a hindgut in which the hook-like shape points leftward (Figure 2; Hozumi et al., 2006). *Myo31DF* mutants are homozygous viable and fertile, suggesting that the role of *Myo31DF* is highly specific for LR asymmetric development (Hozumi et al., 2006; Spéder et al., 2006). In addition, the chiral hindgut epithelial cells in these mutants are the mirror image of their wild-type counterparts (Taniguchi et al., 2011). The forced expression of wild-type *Myo31DF* in the hindgut epithelium of these mutants rescues both the reversed hindgut rotation and the reversed cell chirality. These observations indicated that the default states of LR asymmetry and cell chirality are the mirror image of wild type, and that *Myo31DF* acts to reverse them to the wild-type direction (Hozumi et al., 2006; Taniguchi et al., 2011).

A genetic mosaic analysis of *Myo31DF* suggested that cell chirality is generated intrinsically in each cell, but is also under some influence of neighboring cells, probably through mechanical force (Hatori et al., 2014). These findings collectively suggest that *Myo31DF* acts as a switch for cell chirality and determines the direction of *Drosophila* hindgut rotation.

## Cell-chirality-driven LR asymmetric morphogenesis in various *Drosophila* organs

In addition to the embryonic hindgut, *Myo31DF* mutants in *Drosophila* show LR inversion in various other organs, including the male genitalia, testes, and adult gut, indicating that *Myo31DF* determines the LR asymmetry in these organs, as well (Hozumi et al., 2006; Spéder et al., 2006). Among these organs, cell chirality is known to contribute to the LR asymmetric development of the male genitalia and adult gut (González-Morales et al., 2015; Sato et al., 2015a). Thus, cell chirality appears to be a common strategy for driving the LR asymmetric morphogenesis of tissues in *Drosophila*.

**Figure 4:**
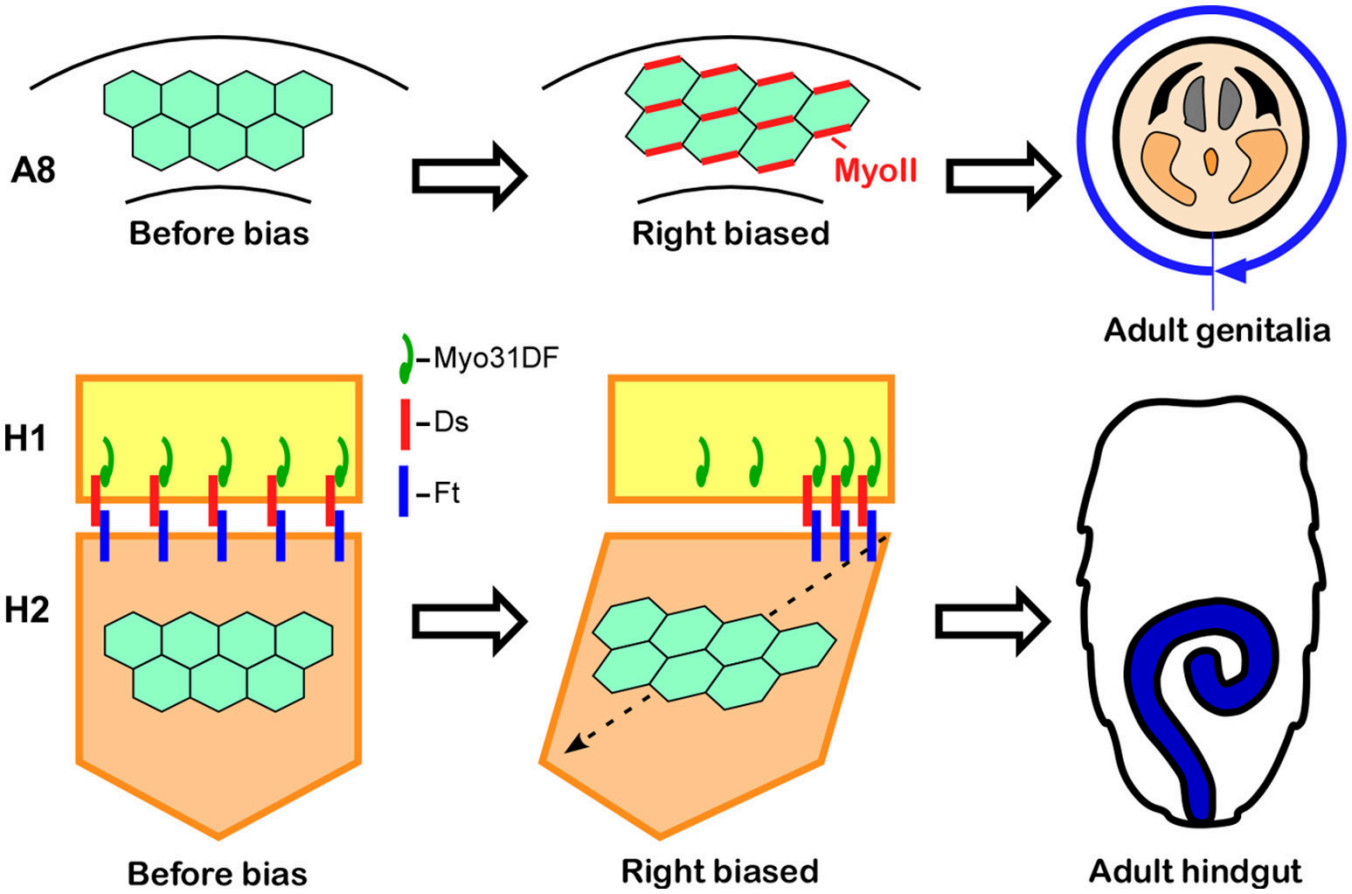
Cell chirality drives LR asymmetric morphogenesis in various *Drosophila* organs. **(Top)** In wild-type *Drosophila* males, the genitalia rotate clockwise as viewed from the posterior. Just before rotation begins, genital epithelial cells in the A8 segment show a chiral cell shape and an asymmetric Myosin II (MyoII) distribution **(Middle)**. This cell chirality drives the 360° rotation of the genitalia **(Right)**. **(Bottom)** The wild-type adult *Drosophila* gut develops from the H2 segment during the pupal stage, while gut laterality is determined by *Myo31DF* expressed in the H1 segment during the larval stage **(Middle)**. Cell chirality is observed only in the H2 segment after the H1 segment is eliminated during metamorphosis. The handedness determined by *Myosin31DF* in the H1 segment is propagated to the H2 segment, leading to the LR-directional looping of the adult hindgut **(Right)**. The atypical cadherins Dachsous (Ds) and Fat (Ft) are thought to be involved in this process. Dachsous is reported to bind *Myo31DF* **(Middle)**. These figures are partly adapted from Inaki et al. (2016) with permission.

The male genitalia in *Drosophila* undergo a 360° clockwise rotation as viewed from the posterior during the pupal stage, achieved by a combination of 180° rotations in two adjacent segments, A8a and A8p (Suzanne et al., 2010; Kuranaga et al., 2011). Just before the rotation begins, A8a epithelial cells show cell chirality (Figure 4; Sato et al., 2015a). In this tissue, Myosin II (MyoII) is concentrated along the rightward tilting cell boundaries (Figure 4; Sato et al., 2015a). This chiral distribution of MyoII is maintained during the rotation and appears to drive LR asymmetric cell intercalation (Sato et al., 2015a). A computer simulation suggested that these LR asymmetric cell interactions give rise to the directional rotation of the male genitalia (Sato et al., 2015a). The cell chirality in the male genitalia is also inverted in *Myo31DF* mutants, consistent with the idea that cell chirality drives the LR directional rotation of this organ (Sato et al., 2015a).

Although cell chirality is also responsible for the LR asymmetric development of the adult gut, the mechanism by which *Myo31DF* and cell chirality contribute to the LR asymmetric morphogenesis of this organ may be different from that of the embryonic hindgut and male genitalia. *Drosophila* undergoes complete metamorphosis, and the adult gut develops from its primordium during the pupal stage (Robertson, 1936; Fox and Spradling, 2009). The adult gut primordium consists of two segments, H1 and H2 (Murakami and Shiotsuki, 2001; González-Morales et al., 2015). The epithelial cells in H2 proliferate to become the adult gut, while the H1 segment is eliminated during the pupal stage. *Myo31DF* is required only in H1 during late larval stage, and cell chirality is observed only in H2 after H1 is eliminated (Figure 4; González-Morales et al., 2015). To explain these observations, it was proposed that polarity determined by *Myo31DF* in H1 is propagated to H2 as cell chirality (Figure 4; González-Morales et al., 2015). In addition, evidence suggested that this propagation of cell chirality is mediated by the unconventional cadherins, Dachsous and Fat (Figure 4; González-Morales et al., 2015). Dachsous and Fat are components of the planer cell polarity (PCP) pathway (Yang et al., 2002). A connection between MyoID, which is an ortholog of Myo31DF, and the PCP pathway was also found in rat (Hegan et al., 2015). The *MyoID* mutation in rat results in the elimination of PCP in multi-ciliated tracheal and brain-ependymal epithelial cells (Hegan et al., 2015). Therefore, some aspects of the relationship between MyoID and PCP may be conserved between *Drosophila* and mammals.

## Molecular function of Myosin31DF in LR asymmetric development

The biochemical functions of the Myosin I family have been studied extensively, and their roles in membrane tension generation, membrane dynamics, and mechanosignal transduction are well-documented (McConnell and Tyska, 2010). Myo31DF is an F-actin-based motor protein, and therefore is likely to exert its function through the actin cytoskeleton; however, the molecular links between cell chirality and Myo31DF remain largely unknown (Hozumi et al., 2006; Taniguchi et al., 2011). To date, a chiral distribution of Myo31DF has not been observed in the *Drosophila* hindgut epithelial cells (Hatori et al., 2014). It may be necessary to observe the dynamics of Myo31DF's localization or activity to understand the biochemical basis of chirality formation.

Potential insight into the connection between the Myosin I family and cell chirality was obtained from an elegant biochemical study of a mammalian Myosin I family protein (Pyrpassopoulos et al., 2012). In this experiment, Myosin1c, a mouse Myosin I family protein (also known as Myosin IC), powered actin motility on fluid-supported lipid bilayers, and this motility was observed to occur along curved paths in a counterclockwise direction (Pyrpassopoulos et al., 2012). Thus, Myosin I family proteins may have an intrinsic chiral property that causes actin to move in a chiral manner. In addition, the actin cytoskeleton has important roles in the chirality of the hindgut epithelial cells in *Drosophila*. Overexpressing a dominant-negative form of Rho1 (Rho1 N19) or Rac1 (Rac1 N17), which is known to regulate F-actin, causes these epithelial cells to become achiral (Taniguchi et al., 2011). Therefore, F-actin dynamics may be important for the formation of cell chirality though Myo31DF.

The *Drosophila* E-Cadherin (*D*E-cad) may also functionally interact with Myo31DF. In *DE-cad* mutants, epithelial cells of the embryonic hindgut become achiral, and the embryonic hindgut rotates in a random direction or does not rotate (Taniguchi et al., 2011). *DE-cad* is also required for the male genitalia rotation (Petzoldt et al., 2012). Genetic analyses suggested that *DE-cad* functions downstream of *Myo31DF* in both organs (Taniguchi et al., 2011; Petzoldt et al., 2012). Myo31DF physically interacts with β-catenin and forms a complex with *D*E-cad, indicating that Myo31DF might regulate *D*E-cad through a β-catenin/cadherin complex (Petzoldt et al., 2012). In the embryonic hindgut, *D*E-cad chirally localizes to adherens junctions, and in *Myo31DF* mutants, this chiral localization of *D*E-cad is the mirror image of that in wild-type (Taniguchi et al., 2011). However, it is not known how Myo31DF, which does not show a chiral distribution in hindgut epithelial cells, controls the chiral localization of *D*E-cad.

## Upstream regulation of *Myosin31DF* in *Drosophila*

By a genetic screen, the *Abd-B* gene was identified as a dominant enhancer of the *Myo31DF* mutation in *Drosophila* (Coutelis et al., 2013). The knockdown of Abd-B by RNA interference in the male genital discs stops the rotation of genitalia (Coutelis et al., 2013). The *Myo31DF* expression in the male genital discs is almost abolished under this condition, and the no-rotation phenotype is rescued by overexpressing *Myo31DF* (Coutelis et al., 2013). The knockdown of *Abd*-B in the embryonic hindgut also stops the hindgut rotation (Coutelis et al., 2013). These results indicate that *Abd-B* acts upstream of *Myo31DF*.

In *Myo31DF* mutants, the embryonic gut and male genitalia still rotate in the reverse direction, suggesting that a “default” pathway exists that drives the inverse rotation of these organs (Hozumi et al., 2006; Spéder et al., 2006). In contrast, *Abd-B* knockdown results in no-rotation phenotypes in these organs. Therefore, the default pathway of LR asymmetric development is also under the control of *Abd-B* (Coutelis et al., 2013). Further experiments are needed to uncover this default pathway. In any case, these results indicate that *Abd-B* may be a master controller of LR asymmetric development in *Drosophila*.

## Roles of other type I myosins in LR asymmetric development in *Drosophila*

*Drosophila* has three type I myosin family proteins: Myo31DF (also known as MyoID), Myo61F (also known as MyoIC), and Myo95E (most similar to MyoIB in vertebrates) (Tzolovsky et al., 2002; Okumura et al., 2015). In addition to *Myo31DF*, potential roles of the other two Myosin I family proteins in LR asymmetric development have been investigated in *Drosophila*. *Myo61F* was proposed to have antagonistic functions to *Myo31DF*, because overexpressing *Myo61F* causes LR inversion of the embryonic gut and male genitalia, reminiscent of the *Myo31DF* loss-of-function phenotypes (Hozumi et al., 2006). Furthermore, these inversion phenotypes induced by *Myo61F* overexpression are suppressed by additionally overexpressing *Myo31DF* (Hozumi et al., 2006; Petzoldt et al., 2012). *Myo61F* inhibits the physical interaction between Myo31DF and the β-catenin/DE-cadherin complex, suggesting how Myo61F may antagonize Myo31DF (Petzoldt et al., 2012). However, the null *Myo61F* mutant alone is homozygous viable and does not show LR defects in the embryonic hindgut or genital disc (Okumura et al., 2015). Thus, the LR-reversing activity of Myo61F overexpression may not reflect its physiological function (Okumura et al., 2015). On the other hand, the *Myo61F* null mutant recessively enhances the clockwise (wild type) genitalia rotation of the *Myo31DF* mutant, suggesting that *Myo61F* and *Myo31DF* have a redundant function in promoting the dextral LR rotation of the male genitalia (Okumura et al., 2015).

Regarding Myo95E, a null mutant of *Myo95E* is homozygous viable and does not show any detectable LR defects (Okumura et al., 2015). In addition, *Myo95E* overexpression results in no obvious LR defects (Okumura et al., 2015). *Myo95E* does not enhance the phenotype of Myo31DF, suggesting that *Myo95E* is not involved in LR asymmetric development (Okumura et al., 2015). Unexpectedly, triple homozygotes for *Myo31DF, Myo61F*, and *Myo95E* lacking their maternal contributions are viable and fertile, and show no obvious developmental defects, except for the LR defects due to the *Myo31DF* mutation (Okumura et al., 2015). Therefore, none of the Myosin I family genes are essential for viability in *Drosophila* (Okumura et al., 2015).

## Chirality of blastomeres is an evolutionarily conserved mechanism for breaking the LR symmetry

In addition to *Drosophila*, the mechanisms of LR asymmetric development have been well studied in other invertebrate species, including air-breathing snails (Pulmonata) and *Caenorhabditis elegans* (*C. elegans*), which are model organisms used to study developmental biology in Ecdysozoa and Lophotrochozoa, respectively (Inaki et al., 2016). In Pulmonata and *C. elegans*, the LR asymmetry of blastomeres at the very early stages of embryogenesis plays central roles in the subsequent LR asymmetric development (Wood, 1991; Bergmann et al., 2003; Shibazaki et al., 2004). These LR asymmetric blastomeres are chiral, given that their shape cannot be superimposed onto its mirror image. In these cases, the chirality of the blastomeres defines the LR asymmetric axis of the next blastomere cleavage, which consequently introduces LR asymmetry into the arrangement of blastomeres (Wood, 1991; Bergmann et al., 2003; Shibazaki et al., 2004). Strikingly, in *Lymnaea stagnalis* (a Pulmonata species) and *C. elegans*, reversing the LR asymmetry in the arrangement of blastomeres by artificial manipulations inverses the entire subsequent LR asymmetric development (Wood, 1991; Kuroda et al., 2009). These results suggest that LR asymmetry in the relative position of blastomeres contains all of the information for the LR axis polarity in the subsequent development.

In Pulmonata, the initial LR asymmetry is observed as cell chirality of the blastomeres at the four-cell stage (Shibazaki et al., 2004). This chirality leads to LR slanting of the cleavage axes of the four blastomeres, which results in a helical shift of the micromeres that are generated by the next cleavage, in a particular direction (Shibazaki et al., 2004). This LR asymmetric rearrangement of macromeres is fully responsible for the entire LR asymmetric development that subsequently occurs, including the coiling direction of the shell and the laterality of the visceral organs (Shibazaki et al., 2004). Notably, in *L. stagnalis*, a maternal *D* mutant that dominantly determines the clockwise (wild type) coiling of the shell in the offspring was identified in a natural population (Pelseneer, 1920; Rolan-Alvarez and Rolan, 1995). In this mutant embryo, the chirality of blastomeres at the four-cell stage disappears, and the blastomeres become radially symmetric, which subsequently leads to the reversed LR asymmetry in the configuration of the eight-cell-stage blastomeres (Shibazaki et al., 2004). Recently, the *D* gene of *L. stagnalis* was found to be a homolog of *formin* (Davison et al., 2016). Formin belongs to a group of proteins that associate with the barbed end of actin filaments and are involved in actin polymerization (Pruyne et al., 2002). These results demonstrate that the actin cytoskeleton is important in the formation of blastomere chirality in this snail.

Actin also plays an important role in the blastomere chirality in *C. elegans*. In this organism, the initial LR asymmetry is detected as a chiral rotation of the actomyosin cortex in the one-cell-stage embryo (Naganathan et al., 2014). This chiral flow is created by turning forces that are generated by myosin under the control of Rho GTPase signaling (Naganathan et al., 2014). In the *C. elegans* embryo, handedness is determined at the four-cell stage (Wood, 1991). The chiral turning force in these blastomeres probably causes a LR asymmetric skewing of the mitotic spindle direction at the four-cell stage, which is responsible for the subsequent LR asymmetric development of the entire body (Wood, 1991; Bergmann et al., 2003).

In summary, the chirality of blastomeres plays critical roles in the LR asymmetric development of these two representative model organisms of Ecdysozoa and Lophotrochozoa. Given that the chirality of blastomeres is a specific version of cell chirality found during early cleavage stages, cell chirality may have general and evolutionarily conserved roles in LR asymmetric development.

## Cell chirality in vertebrates

**Figure 5:**
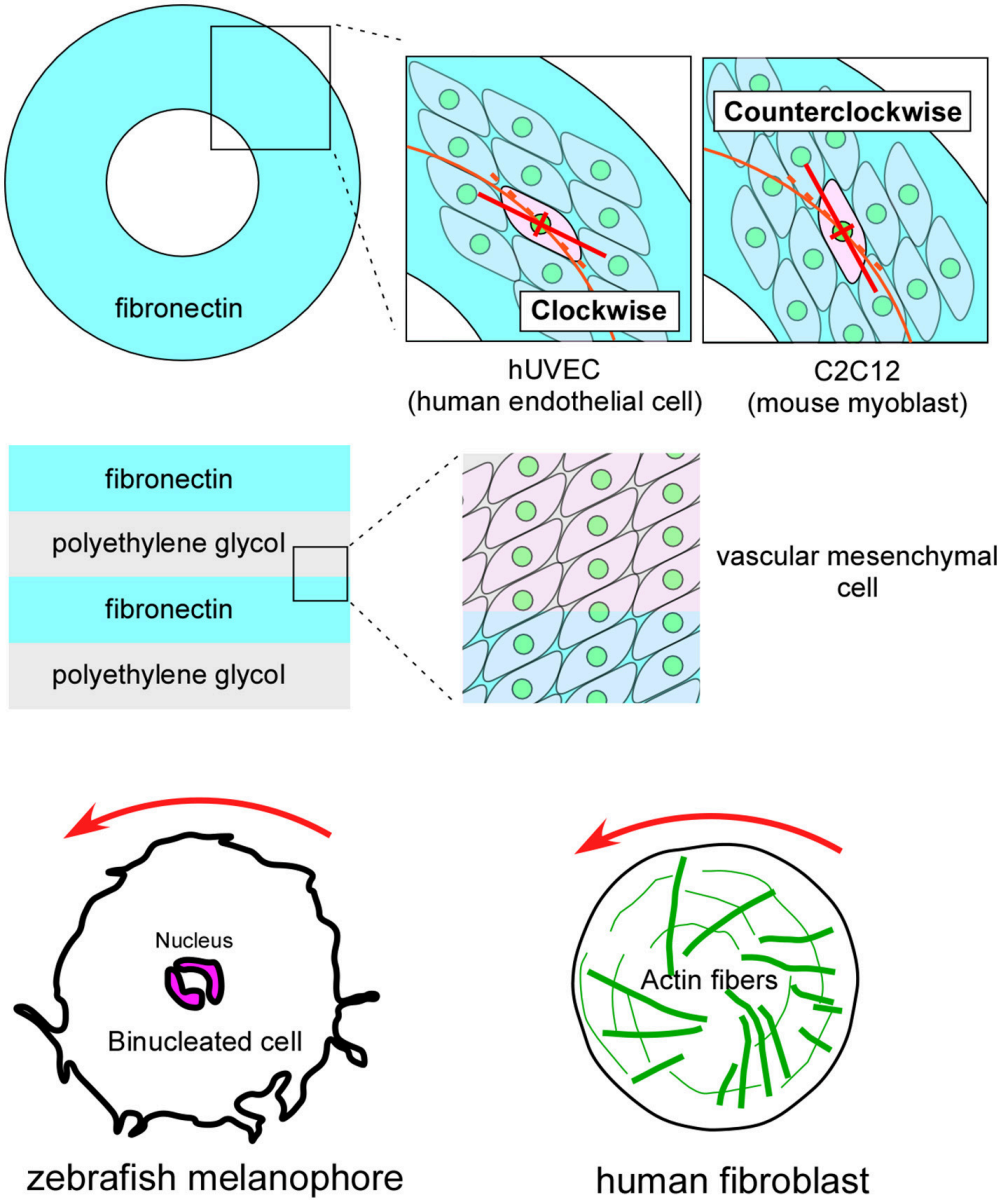
Vertebrate cultured cells exhibit intrinsic cell chirality. **(Top)** Cultured human endothelial (hUVEC) and mouse myoblast (C2C12) cells are arranged in clockwise and counterclockwise spiral patterns on a substrate (Fibronectin) with a ring micropattern (Wan et al., 2011). **(Middle)** Vascular mesenchymal cells exhibit intrinsic chirality when plated on a substrate with a stripe micropattern (Fibronectin and polyethylene glycol) (Chen et al., 2012). **(Bottom)** A counterclockwise rotation of the nucleus is observed in cultured zebrafish melanophores **(Left)** (Yamanaka and Kondo, 2015). Human fibroblasts cultured on a micropattern substrate show chiral swirling **(Right)** (Tee et al., 2015). These figures are partly adapted from Inaki et al. (2016) with permission.

Although cell chirality and blastomere chirality have been found *in vivo* only in invertebrates, many vertebrate cells demonstrate various indications of cell chirality under certain culture conditions (Figure 5; Xu et al., 2007; Wan et al., 2011; Chen et al., 2012; Tee et al., 2015; Worley et al., 2015; Yamanaka and Kondo, 2015; Raymond et al., 2016). Cell lines including mouse myoblasts (C2C12), human umbilical vein endothelial cells (hUVEC), and canine kidney epithelial cells (MDCK) exhibit a chiral cell shape when cultivated on ring or stripe micro-patterns of adhesive substrates (Figure 5; Wan et al., 2011; Chen et al., 2012; Worley et al., 2015; Raymond et al., 2016). Cell chirality is also found in cellular behaviors under certain culture conditions. For example, human blood cells (dHL60) show leftward biased directional movement in the absence of directional chemotactic cues (Xu et al., 2007). Cell chirality is also observed in the dynamics of intracellular structures. Cultured zebrafish melanophores show a counterclockwise rotation of their dinuclei and cytoplasm (Figure 5), and the actin cytoskeleton plays a critical role in this chiral behavior (Tee et al., 2015; Yamanaka and Kondo, 2015). Human foreskin fibroblasts seeded on a circle micro-pattern show a chiral swirling of actin fibers (Figure 5; Tee et al., 2015). Intriguingly, in addition to the appropriate actin cytoskeleton structure, Formin activity appears to be required to generate this chiral swirling of actin fibers; similarly, the *formin* gene has an essential role in creating blastomere chirality in a snail (Davison et al., 2016). Although the biological relevance of the vertebrate cell chirality observed *in vitro* remains elusive, many cell lines from various organs show distinct chirality (Wan et al., 2011), suggesting that cell chirality might drive the LR asymmetric morphogenesis of these organs, as in invertebrates.

**Figure 6:**
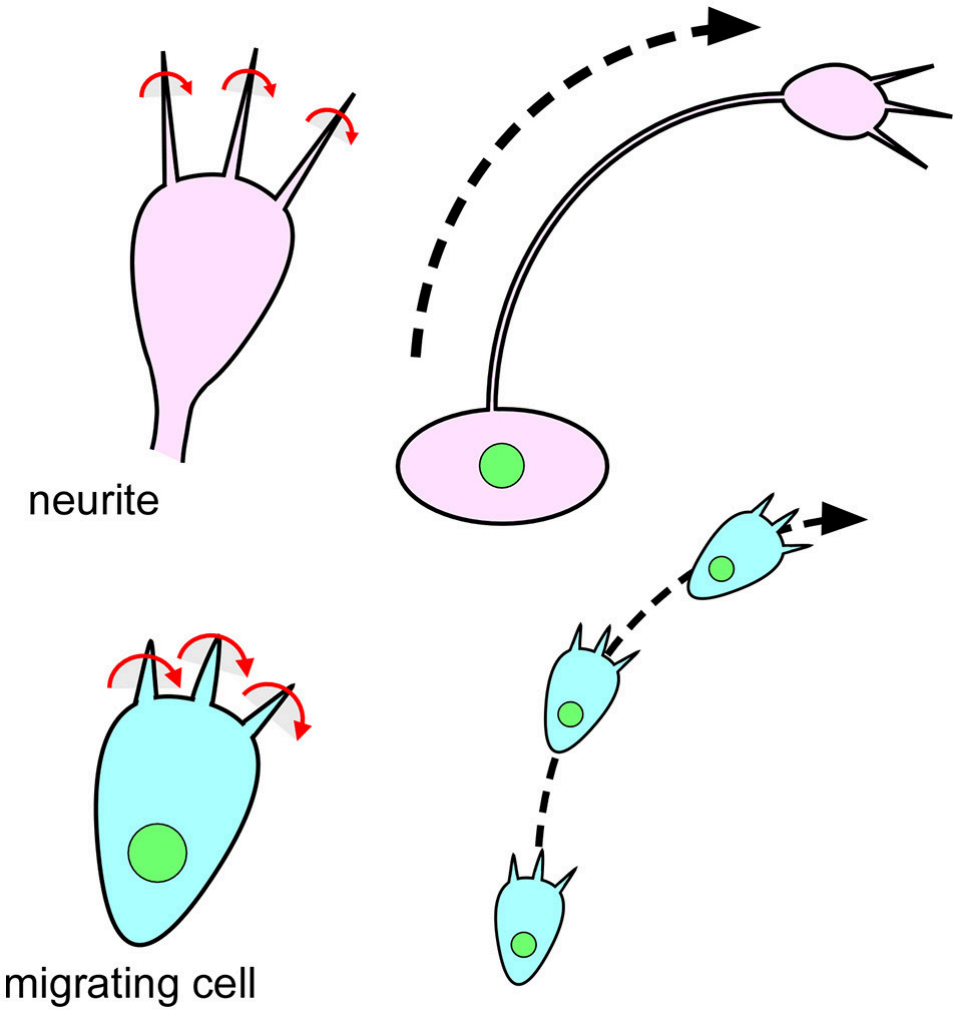
The chiral behavior of filopodia is evolutionarily conserved from single-celled eukaryotic organisms to mammals. **(Top)** Filopodia of the growth cone on mouse neurites rotate in a rightward screwlike manner **(Left)** (Tamada and Igarashi, 2017). The right-screw rotation of filopodia introduces a clockwise growth of neurites **(Right)** (Tamada and Igarashi, 2017). **(Bottom)** Filopodia of *Dictyostelium* cells also show a rightward-screwlike rotation **(Left)** (Tamada and Igarashi, 2017). The right-screw rotation of filopodia leads the cells to migrate in a clockwise direction **(Right)** (Tamada and Igarashi, 2017).

In addition to these vertebrate cells, chiral cellular behaver was recently reported in *Dictyostelium discoideum* (Tamada and Igarashi, 2017). During their ameboid movement, *Dictyostelium* cells extend filopodia that rotate in a rightward screwlike manner (Figure 6; Tamada and Igarashi, 2017). This right-screw rotation is very similar to the movement of filopodia on the growth cone of mouse neurons (Figure 6; Tamada et al., 2010; Tamada and Igarashi, 2017). It was proposed that the right-screw rotation of filopodia introduces the observed clockwise tracks in neurite growth and in cell migration on two-dimensional substrates (Figure 6; Tamada and Igarashi, 2017). These findings suggest that cell chirality can be traced back to single-celled eukaryotic organisms.

## Concluding remarks

Today, it is well-accepted that many animal cells show intrinsic cell chirality. Although the molecular mechanisms of cell chirality formation are still largely unknown, F-actin appears to play critical roles in this process, as revealed in *Drosophila*, snails, *C. elegans*, and vertebrates. In particular, the essential role of Formin family proteins in the cell chirality formation of snail blastomeres and mammalian cells suggests that the axial rotation of F-actin may be one of the origins of chirality (Tee et al., 2015; Davison et al., 2016). In *Drosophila*, Myo31DF may switch the chiral status of the F-actin structure or dynamics, although this possibility remains to be proven.

In *Drosophila*, several lines of evidence show that cell chirality is a common mechanism in this organism for developing LR asymmetric structures. On the other hand, in vertebrates, although cell chirality is broadly found, its functions remain a mystery. A computer simulation predicted that a model cell with cell chirality may have an advantage for passing through a population of cells (Sato et al., 2015b). In addition, it is possible that cell chirality interferes or collaborates with PCP, thereby disrupting or modifying the architecture of the planarly polarized cells. This could occur in two ways: cell chirality itself may be superimposed onto the PCP, or chiral cells may intermingle with PCP-exhibiting cells. By such processes, cell chirality may control various morphogenetic events, not only LR asymmetric development. The investigation of cell chirality is still in its infancy, and many questions, especially about the molecular mechanisms of its formation and biological functions, are still open. Answering these questions will add valuable insight for studies in biology and medicine in the near future.

## Author contributions

MI, TS, and KM wrote the review; MI and TS made figures.

### Conflict of interest statement

The authors declare that the research was conducted in the absence of any commercial or financial relationships that could be construed as a potential conflict of interest.
